# Three Weeks on a Ketogenic Diet Reduces Free Testosterone and Free Estradiol in Middle-Aged Obese Men and Women

**DOI:** 10.1155/2024/9301369

**Published:** 2024-08-06

**Authors:** Mads Svart, Nikolaj Rittig, Thien Vinh Luong, Nigopan Gopalasingam, Esben Thyssen Vestergaard, Lars Gormsen, Esben Søndergaard, Henrik Holm Thomsen, Claus H. Gravholt

**Affiliations:** ^1^ Department of Internal Medicine Horsens Regional Hospital, Horsens, Denmark; ^2^ Department of Internal Medicine and Endocrinology Aarhus University Hospital, Aarhus, Denmark; ^3^ Steno Diabetes Center Aarhus Aarhus University Hospital, Aarhus, Denmark; ^4^ Department of Nuclear Medicine & PET-centre Aarhus University Hospital, Aarhus, Denmark; ^5^ Department of Cardiology Aarhus University Hospital, Aarhus, Denmark; ^6^ Department of Cardiology Gødstrup Hospital, Herning, Denmark; ^7^ Department of Pediatrics Aarhus University Hospital, Aarhus, Denmark; ^8^ Department of Clinical Medicine Aarhus University Hospital, Aarhus, Denmark; ^9^ Department of Internal Medicine Viborg Regional Hospital, Viborg, Denmark; ^10^ Department of Molecular Medicine Aarhus University Hospital, Aarhus, Denmark

## Abstract

**Background:**

Beta-hydroxybuturate (*β*-OHB) supplements are commonly utilized in sports by both recreational and professional athletes. In a recent study, we observed a drop in testosterone levels following the oral ingestion of racemic sodium-*β*-OHB. In this investigation, we aim to determine whether a single oral dose of ketone ester (study I) and prolonged endogenous ketosis (study II) also reduces testosterone levels.

**Design:**

This investigation integrated samples from two distinct studies. Study I was a randomized, controlled, crossover trial with ten healthy, young male participants receiving either a weight-adjusted ketone ester or control (water, CTR) and vice versa following an overnight fast. Repeated blood sampling was used to monitor plasma *β*-OHB and testosterone levels. Study II, another randomized, controlled, crossover trial, included 11 middle-aged participants (five males). They followed either a ketogenic diet (KD) characterized by low carbohydrates and high fat content or a standard diet (SDD) for three weeks. After each study period, participants underwent examination following an overnight fast, with repeated measures employed to analyze concentrations of plasma *β*-OHB and sex hormone levels.

**Results:**

Study I: Testosterone decreased from 23.8 ± 2.4 nmol/l to 22.3 ± 2.5 nmol/l 300 minutes after the ketone ester and increased from 20.9 ± 2.1 nmol/l to 22.2 ± 1.9 300 minutes after CTR. This difference was not significant, *p* = 0.06. *Study II*. Total testosterone was unaffected after the KD compared to the SDD in men (20.2 ± 1.23 nmol/l vs. 18.2 ± 1.23 nmol/l (*p* = 0.1)) and was lower after KD in women (0.87 ± 0.06 vs. 1.1 ± 0.06 nmol/l (*p* < 0.0001)). Sex hormone-binding globulin (SHBG) increased in men after KD compared with SDD (31.2 ± 2.6 nmol/l vs 25.0 ± 2.6 nmol/l, *p* < 0.0001) and women (26.5 ± 3.05 nmol/l vs 24.2 ± 3.05 nmol/l, *p* = 0.003). The free androgen index decreased after KD in men (ratio: 0.65 ± 0.05 vs. ratio: 0.74 ± 0.05, *p* = 0.04) and in women (ratio: 0.036 ± 0.006 vs. SDD 0.05 ± 0.006, *p* = 0.0001). Free estradiol index was also found lower after KD in men (ratio: 3.1 ± 0.8 vs. ratio: 4.8 ± 0.8, *p* = 0.0003) and in women (ratio: 1.2 ± 2.2 vs. 9.8 ± 2.2, *p* = 0.0001).

**Conclusion:**

Our findings indicate that the acute ingestion of ketone ester may not reduce testosterone levels in healthy young males. However, a three-week exposure to KB from a KD results in an increase in SHBG in men and women with obesity as well as it lowers free testosterone and estradiol for men and women. We thus present evidence of crosstalk between alterations in a metabolite, *β*-OHB, and the regulation of the hypothalamic-pituitary-gonadal axis from a KD. The clinical impact of this reduction remains to be investigated. This trial is registered with NCT04156477 and NCT05012748.

## 1. Background

Endogenous ketone bodies are produced during times of diabetic ketoacidosis, fasting, low-carbohydrate diet, and bodily stress, e.g., infection or strenuous exercise [1]. The ketone body, *β*-hydroxybuturate (*β*-OHB), is being used as a supplement in sports athletes in an attempt to improve performance [2, 3]. However, it is conflicting whether *β*-OHB supplements improve physical performance [4], psychological performance [3], or postexercise recovery [5].

Ketone supplements are mainly available as ketone salts or ketone esters, and both approaches result in an elevated circulating concentration of *β*-OHB ranging from 0.5 mmol/l to 5.5 mmol/l [6, 7]. Many of these approaches lead to weight loss which in turn raise testosterone [8]. Exogenous ketosis is obtainable through various methods of which infusion of racemic sodium-*β*-OHB (salt) and ingestion ketone esters are frequently employed [9]. Sustained intake of *β*-OHB does not consistently result in weight loss, making it a suitable option for maintaining ketosis.

Testosterone improves physical performance [10], maintains skeletal muscle mass and strength on top of its key functions on libido and sexual function, mood, cognition, and cardiovascular health [11, 12]. A potential testosterone decline may counteract some of the potential positive effects associated with ketosis [13].

Recently, we showed that an oral sodium-*β*-OHB dose decreased testosterone in healthy young males [14]. To further investigate whether ketosis is associated with lower testosterone levels, we aimed to investigate if this finding is compound specific and if longer lasting endogenous ketosis also lowers testosterone.

Consequently, we examined the effects of a three-week ketogenic diet (KD) on testosterone and estradiol concentrations in samples from middle-aged, obese men and women, as well as the effects of a single oral ketone ester dose on testosterone concentrations in samples from healthy young males. We hypothesized that testosterone concentrations would decrease following acute ketosis induced by ketone ester intake and longer-lasting endogenous ketosis from KD.

## 2. Methods

### 2.1. Study Design

We used samples from two randomized, controlled trials, whereof other data have previously been published [15, 16].

#### 2.1.1. Acute Ketone Ester Ingestion

In brief, the study included ten healthy young males investigated on three separate occasions of which we report data from the placebo (CTR) and ketone ester ingestion (KET) study days. In this study, the participants acutely ingested 714 mg/kg ketone monoester D-BHB-R-1,3-butanediol (HVMN V1 ketone ester) or taste-adjusted water and underwent blood sampling for five hours [7, 16].

#### 2.1.2. Ketogenic Diet

In brief, the study included 11 (five males, six females) healthy, overweight participants. All participants received an individualized KD plan adjusted for calorie intake based on sex, weight, and approximate activity level. Each diet plan included options for every meal, ensuring a macronutrient distribution of 5 E% carbohydrates, 20 E% protein, and 75 E% fat. These diet plans were created by a dietitian using Vitakost Pro, a professional dietary tool that utilizes the Danish Food database. A low glycemic index source of carbohydrate was recommended during the KD. Fat intake ranged from 200 to 300 grams/day, with an even distribution between saturated and monounsaturated fatty acids. Participants were responsible for buying ingredients, preparing meals, and adhering to the diet in their usual environment. During the standard diet (SDD), participants were instructed to eat with a macronutrient distribution following the Nordic Nutritional Recommendations (45–60 E% carbohydrates, 10–20 E% protein, 25–40 E% fat). The participants were randomly assigned in a 1 : 1 ratio to receive either KD or SDD in the first study period. To ensure compliance, all participants were instructed to measure their blood *β*-OHB concentration using a point-of-care ketonometer (Freestyle Libre; Abbott Diabetes Care Ltd., England) every morning (7: AM) and evening (7: 00 PM) during both interventions. Results were reported daily. Participants measured *β*-OHB twice daily and those who failed to increase their plasma ketone levels to 0.3 mmol/l during the first week of KD were excluded from the study. An experimental day was included at the end of each trial period [15].

### 2.2. Hormones Analysis

Androstenedione, testosterone, estradiol, and dehydroepiandrostenedione sulphate (DHEAS) were measured using an in-house LCMS-MS assay (Sciex, Framingham, MA, USA). The assays were calibrated using a commercially available 6-point calibration curve (Chromsystems, Gräfelfing, Germany). The assay was calibrated using a 3-point calibration curve made from charcoal-stripped fetal bovine serum (Sigma-Aldrich, St. Louis, MO, USA) and spiked with weighed-in pure compounds (Toronto Research Chemicals and Steraloids, Toronto, Canada). FSH, LH, and SHBG were measured using automated electrochemiluminescence assays on the Cobas 8000 analyzer, e-module (Roche Diagnostics, Copenhagen, Denmark).

### 2.3. Ethics

Study I was conducted after approval by the local ethics committee (#1-10-72-7-19) and registered at https://www.clinicaltrials.gov (NCT04156477) before enrolment. Study II was conducted after approval by the local ethics committee (#1-10-72-232-18) and registered at https://www.clinicaltrials.gov (NCT04156477). Before enrolment all participants gave oral and written informed consent.

### 2.4. Statistical Analysis

A linear mixed model was used to compare the effects of ketone ester treatment compared with placebo and KD with SDD. Treatment, period, and treatment sequence were defined as fixed effects and patients as random effects. For the repeated measurements during the sampling period after either a single dose KE/placebo treatment or KD, a treatment-by-time interaction was added as fixed effects. The reported *p* values were calculated using least-squares means analyses of the respective linear mixed models. The residuals were tested for normality and homoscedasticity. Data were log transformed if needed. The effect size of KE treatment compared with placebo treatment is presented as pairwise mean difference with SEM.

All graphics and statistical analyses were performed using SigmaPlot 14 (San Diego, CA, USA) and R (Version 2022.12.0, RStudio, Posit, USA).

## 3. Results

### 3.1. Study 1 (Oral 3OHB Ester Ingestion)

Ketosis was achieved and the participants reached a peak ketone concentration around 5.5 mmol/l.

**Figure 1:**
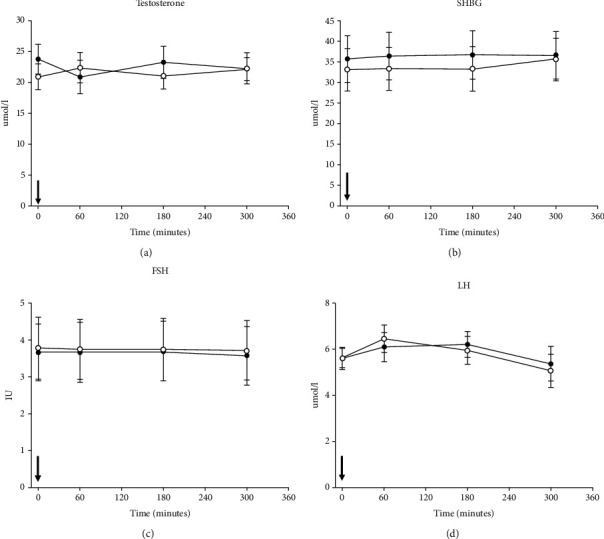
Data from all ten participants in study 1. Panel (a) total testosterone, (b) sex hormone binding globulin (SHBG), (c) Follicle stimulating hormone (FSH), and (d) Luteinizing hormone (LH) concentrations are shown on the *y*-axis and time on the *x*-axis. The black arrow at time = 0 minutes indicates the time of ingesting either ketone ester (KET) or placebo (CTR). Black circles illustrate the mean value (±SEM) on the KET day and white circles illustrate the mean value (±SEM) on the CTR day. Linear mixed model was used to test for between-treatment pairwise comparison (KET/CTR).

Total testosterone concentrations dropped during KET (Baseline: 23.8 ± 2.4 nmol/l to 22.3 ± 2.5 nmol/l) and increased during CTR (Baseline: 20.9 ± 2.1 nmol/l to 22.2 ± 1.9 nmol/l) after an acute ingestion of the ketone ester drink in healthy young males. However this finding was not statistically significant, *p* = 0.06. SHBG increased in KET 36.2 ± 5.82 vs CTR 33.2 ± 5.82, *p* = 0.002 (Figure 1(b)). A slight decrease in FSH was observed in KET 3.72 ± 0.87 vs 3.82 ± 0.87, *p* = 0.04 (Figure 1(c)). No change was observed in LH (Figure 1(d)), androstenedione, 17-hydroxyprogesterone, or in DHEAS (data not shown).

### 3.2. Study 2 (Ketogenic Diet)

Eleven (five males, six females, post-menopausal) healthy, overweight participants with body mass index (BMI): 28–40 kg/m^2^ and age between 50 and 75 years were included. Three weeks of KD induced a significant higher circulating *β*-OHB concentrations compared to a standard diet (SDD) (1.0 ± 0.1 mmol/l vs. 0.1 ± 0.01 mmol/l), as previously reported [15]. We measured sex hormones at three time points after each of the three weeks intervention. There was no carry-over effect detected and no interaction of visit order.

### 3.3. Testosterone

**Figure 2:**
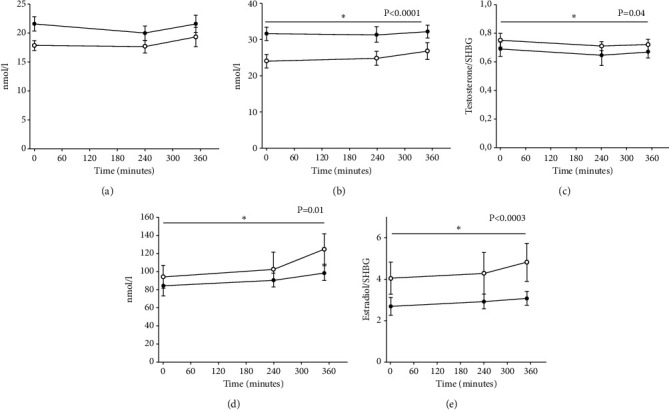
Hormone levels for five male participants in study 2. Panel (a) Total testosterone, (b) sex hormone binding globulin (SHBG), (c) free androgen index (FAI) (total testosterone/SHBG), (d) total estradiol, and (e) free estradiol index (FEI) (total estradiol/SHBG). Black circles illustrate the mean value (±SEM) on the KET day and white circles illustrate the mean value (±SEM) on the CTR day. Linear mixed model was used to test for between-treatment pairwise comparison (KET/CTR).

**Figure 3:**
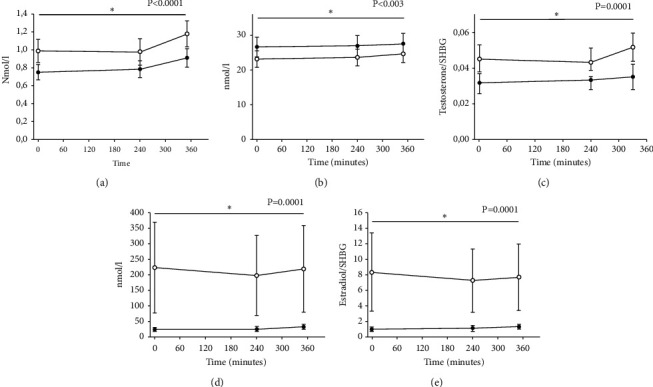
Hormone levels for six female participants in study 2. Panel (a) total testosterone, (b) sex hormone binding globulin (SHBG), (c) free androgen index (FAI) (total testosterone/SHBG), (d) total estradiol, and (e) free estradiol index (FEI) (total estradiol/SHBG). Black circles illustrate the mean value (±SEM) on the KET day and white circles illustrate the mean value (±SEM) on the CTR day. Linear mixed model was used to test for between-treatment pairwise comparison (KET/CTR). Unequally distributed data (panel (d)) was log transformed for statistics. Data in graphs are actual values and not log transformed.

There was no difference in total testosterone during the six hour observation period after KD vs. SDD in men (20.2 ± 1.2 nmol/l vs. 18.2 ± 1.2 nmol/l, *p* = 0.1) (Figure 2(a)). However, testosterone was lower in women after KD vs. SDD (0.9 ± 0.06 vs. 1.1 ± 0.06 nmol/l (*p* < 0.0001)) (Figure 3(a)). SHBG was higher in men over time after KD vs. SDD (31.2 ± 2.6 nmol/l vs. 25.0 ± 2.6 nmol/l, *p* < 0.0001) (Figure 2(b)), and the same change in SHBG was present in women (KD vs. SDD: 26.5 ± 3.1 nmol/l vs. 24.2 ± 3.1 nmol/l, *p* = 0.003) (Figure 3(b)). We calculated a free androgen index (FAI) using total testosterone and SHBG to correct for different binding capacity [17]. The FAI for men was lower after KD vs SDD (ratio: 0.65 ± 0.05 vs. ratio: 0.74 ± 0.05, *p* = 0.04) (Figure 2(c)). The FAI for women was lower too after KD ratio: 0.036 ± 0.006 vs. SDD 0.05 ± 0.006 (*p* = 0.0001, Figure 3(c)).

### 3.4. Estradiol

We found lower estradiol in men KD vs SDD (97 ± 16 nmol/l vs. 117 ± 16 nmol/l, *p* = 0.01) and women after KD vs SDD (28.3 ± 70 nmol/l vs. 279 ± 70 nmol/l, *p* = 0.0001) (Figure 2(d)*+*3(d)).

We calculated the free estradiol index (FEI) [18], with lower FEI in men after KD vs. SDD (ratio: 3.1 ± 0.8 vs. ratio: 4.8 ± 0.8, *p* = 0.0003) (Figure 2(e)) and women KD vs SDD (ratio: 1.2 ± 2.2 vs. 9.8 ± 2.2, *p* = 0.0001) (Figure 3(e)). LH and FSH wERE unaltered in the two conditions for men and women (data not shown).

## 4. Discussion

We investigated the effects of acute ketosis on human sex hormone regulation in men and women. We hypothesized that acute ketone ester ingestion would lower testosterone. However, this was not the case from drinking ketone ester contrary to our previous findings after drinking sodium-*β*-OHB [14]. We further investigated the effect of endogenous prolonged KD-induced ketosis on sex hormones in middle-aged, obese men, and women. Here, we hypothesized that KD would lower testosterone. This was the case in men and women with increased SHBG and lower FAI for both sexes. We further found lower total estradiol as well as FEI in men and women after three weeks of KD.

### 4.1. Study 1

We found no difference in testosterone from drinking the ketone ester. Whether the effect is different from long term use remains uncertain. It is notable that by chance, the baseline values in the two groups at baseline were higher on the KET day. The finding is contrary to previous findings where we observed with Na-D/L-*β*-OHB ingestion in a similar group of young healthy males [14].

### 4.2. Study 2

During KD, *β*-OHB increased to approximately 1 mmol/l and remained at overnight fasting values around 0.1 mmol/l during the SDD. Notably, total testosterone declined in women and was unaltered in men. Among obese males we also observed a large (approximately 50%) increase in SHBG, and the calculated FAI decreased significantly, following KD. In women an SHBG significant increase of some 10% was also observed together with a significant decline in FAI. Obesity is a well-established risk factor for insulin resistance and diabetes [19]. In postmenopausal women not using hormone replacement therapy, a lower SHBG and a high FAI is associated with cardiovascular events [20]. It is also suggested that high levels of SHBG favors a healthy cardiometabolic profile [21] and testosterone in men is protective with regards to cardiometabolic health [22].

Hydroxycarboxylic acid receptor 2 (HCAR2) is present in hepatocytes [23] and hepatic macrophages promoting anti-inflammation [24]. The age-related loss of HCAR2 in hepatic tissue in rodents is suggested to play an important role in hepatic lipid accumulation underlining the link between *β*-OHB and metabolic health [25]. Hence, it is noteworthy that just three weeks of adopting the KD intervention resulted in elevated SHBG levels in obese men and women, potentially improving their cardiometabolic risk profile. Metabolic associated fatty liver disease (MAFLD) correlates strongly with low levels of SHBG [26]. MAFLD increase with age in men and women [27] and KD is shown to improve MAFLD, and this could link the KB increase to the prominent SHBG increase in our study [28].

A HCAR2 signaling effect cannot be ruled out either, as the pituitary gland and both sex gonadal glands express HCAR2 on their surface [23]. The unchanged LH and FSH after KD point towards a steady state hormone balance. The signaling, if present, most likely takes place in the gonads as the pituitary hormones are completely stable in both studies.

We found lower estradiol in men and women as well total as SHBG corrected FEI. In women, estradiol is a well-established protective factor against cardiovascular disease [29]. On the other hand, low estradiols in women are a protective factor with regards to breast cancer [30]. In men the absence or declining estradiol levels is correlated with sexual dysfunction [31].

One major limitation of our study was the lack of power in the KD study to investigate men and women separately, as it was designed to investigate cardiac metabolism with no obvious need to divide the participants in men and women. Investigating the *β*-OHB effect on sex hormones we needed to split the population into males and females, which left only five men and six women in each group Both studies employed crossover design, with each participant serving as their own control, thereby enhancing the studies' power and the reliability of the results. Carry-over effects were ruled out as was the intervention order.

In conclusion, our findings suggests that consuming ketone esters does not affect testosterone levels acutely in healthy young men, contrary our previous study on Na-D/L-b-OHB. Furthermore, our investigation revealed that a ketogenic diet for three weeks induced higher levels of SHBG in obese men and women. This finding was accompanied by lower FAI and FEI in men and women after KD. This sheds light on a possible interplay between changes in *β*-OHB, a metabolite, and the control of the hypothalamic-pituitary-gonadal axis, over time.

## Data Availability

Data are available upon request, but permission must, in selected cases, first be obtained from the Danish Data Protection Agency.
